# Role of JAK-STAT signaling in maturation of phagosomes containing *Staphylococcus aureus*

**DOI:** 10.1038/srep14854

**Published:** 2015-10-07

**Authors:** Fei Zhu, Yadong Zhou, Chunxia Jiang, Xiaobo Zhang

**Affiliations:** 1 Collaborative Innovation Center of Deep Sea Biology, Key Laboratory of Animal Virology of Ministry of Agriculture and College of Life Sciences, Zhejiang University, Hangzhou 310058, China; 2 College of Animal Science and Technology, Zhejiang Agriculture and Forestry University, Hangzhou 311300, China

## Abstract

Phagocytosis is a required mechanism for the defense against pathogens. *Staphylococcus aureus*, an important bacterial pathogen, can promptly escape from phagosomes and proliferate within the cytoplasm of host. However, the mechanism of phagocytosis against *S. aureus* has not been intensively investigated. In this study, the *S. aureus* was engulfed by macrophages (RAW264.7 cells) but not digested by the cells, suggesting that the phagosomes did not maturate in macrophages. Further investigation revealed that peptidoglycan (PG) induced the phagosome maturation of macrophages, resulting in the eradication of *S. aureus*. Genome-wide analysis and quantitative real-time PCR indicated that the JAK-STAT pathway was activated by PG during the phagosome maturation of macrophages against *S. aureus*. This finding presented that the PG-activated JAK-STAT pathway was required for phagosome maturation. Therefore, our study contributed evidence that revealed a novel aspect of PG-triggered JAK-STAT pathway in the phagosome maturation of macrophages.

*Staphylococcus aureus* is a major human pathogen causing significant morbidity and mortality due to both community- and hospital-acquired infections^1^. This pathogen causes a variety of diseases, including impetigo, cellulitis, food poisoning, toxic shock syndrome, necrotizing pneumonia, and endocarditis. The ability of *S. aureus* to cause disease is based partly on its ability to subvert the innate immune system of hosts. Increasing evidence indicates that *S. aureus* may be a facultative intracellular pathogen^2^. Recent reports suggest that *S. aureus* can persist inside macrophages for several days without affecting the viability of these cells^3^. At present, the interactions between *S. aureus* and host innate immunity become important issues to be addressed.

The innate immunity in vertebrates and invertebrates is of central importance as the first-line defense against pathogenic challenges^4^. In the innate immunity of animals, phagocytosis is one of the most powerful and immediate ways to eliminate pathogens^5^. Phagocytosis, an evolutionarily conserved process, can be utilized by cells to ingest microbial pathogens, and apoptotic and necrotic corpses^6^. Macrophages, possessing phagocytic activity, play a key role in host defense by recognizing, engulfing and killing microorganisms. However, some pathogens can persist inside these cells, evading host defense^7^. As a member of gram-positive bacteria, *S. aureus* can induce a cytoprotective effect in macrophages, which can prevent host cell elimination and thus allow intracellular bacterial survival^8^. In murine macrophages, the expression of CXCR1 (interleukin-8 receptor) induced by *S. aureus* is implicated in the intracellular survival of bacteria via cytokines and bacterial anti-oxidant enzymes^9^. As reported, *S. aureus* has developed many antiphagocytic properties. Protein A, a major cell wall component of *S. aureus*, may cause polysaccharide-independent biofilm formation and prevent macrophage phagocytosis^10^,^11^. Lipoteichoic acid (LTA) and clumping factor A (ClfA), a fibrinogen-binding surface protein of *S. aureus*, can interfere and inhibit the phagocytosis of leucocytes against *S. aureus*^12^. However, the interactions between *S. aureus* and host phagocytosis remain to be characterized.

It is well known that some kinds of polysaccharides from bacteria can activate the phagocytosis of macrophages against bacteria. Lipopolysaccharide (LPS), a major component of the cell walls of gram-negative bacteria, can induce macrophages to phagocytose gram-negative bacteria through the pattern recognition receptor Toll-like receptor 4 (TLR4)^13^. Peptidoglycan (PG), a major component of the cell walls of gram-positive bacteria, plays very important roles in phagocytosis against gram-positive bacteria through Toll-like receptor 2 (TLR2), which is recruited to phagosomes and discriminates between pathogens^14^,^15^,^16^. For phagocytosing *S. aureus*, PG must be particulated and internalized via phagocytosis^17^. The heat-inactivated *S. aureus* is a common model to investigate the phagocytosis process, the phagosome acidification, the bacterial recognition and the cytokine production in response to *S. aureus* in phagocytes^18^,^19^. At present, the role of polysaccharides in the regulation of phagocytosis against *S. aureus* is not clear.

To address this issue, the phagocytosis of *Mus musculus* RAW264.7 cell, a macrophage-derived cell line, was evaluated using *S. aureus* in this study. The results revealed that the PG-triggered JAK-STAT signaling pathway contributed to the phagosome maturation of macrophages against *S. aureus*.

## Results

### Peptidoglycan induces phagocytosis of inactivated *S. aureus* in RAW264.7 cells

**Figure 1:**
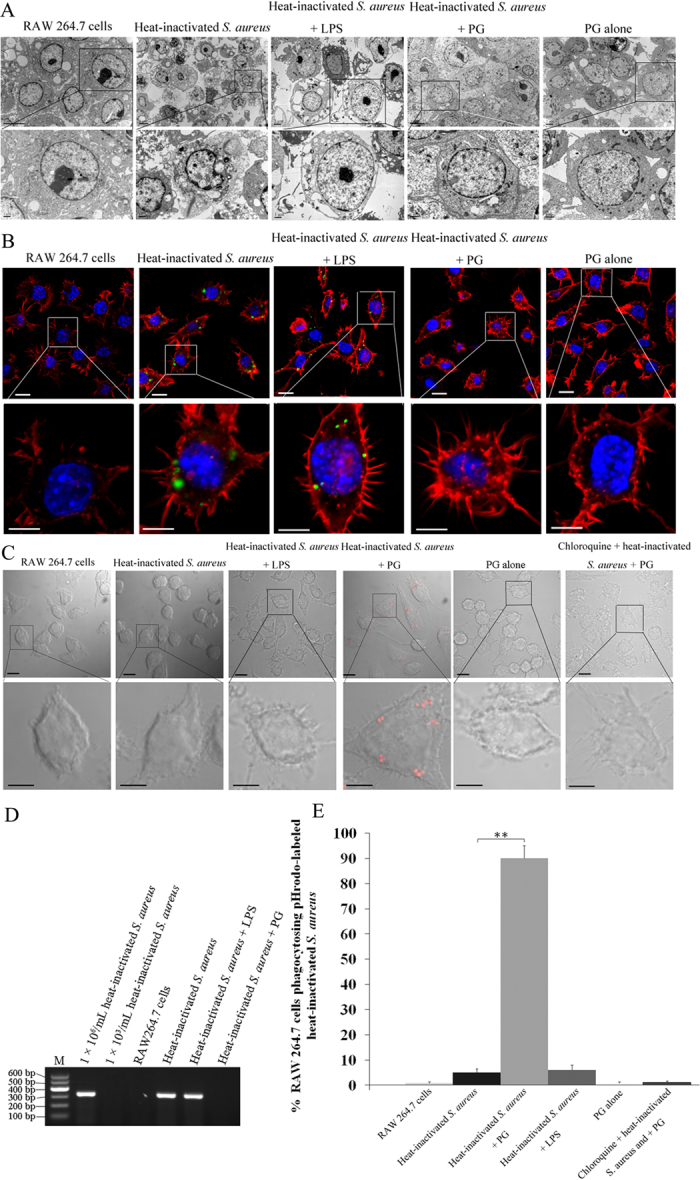
Peptidoglycan induces phagocytosis after inoculation with inactivated *S. aureus*. (**A**) RAW264.7 cells were induced with LPS or PG inoculated with inactivated *S. aureus* and inactivated *S. aureus*. One day later, the cells were examined with transmission electron microscope. RAW264.7 cells only and PG alone were used as controls. Lane headings indicated the treatments. The box indicated the enlarged image. Scale bar, 5 μm (up) or 1 μm (down). (**B**) RAW264.7 cells were treated with LPS or PG and then with FITC-labeled inactivated *S. aureus*. After incubation for 1 day, the cells were subjected to phagocytosis assays. The filamentous actin was stained with rhodamine phalloidin and the cellular DNA with DAPI. The green spots indicated the internalized *S. aureus*. Non-treated cells and PG alone were used as controls. Scale bar, 10 μm (up) or 5 μm (down). (**C**) RAW264.7 cells were incubated with LPS or PG and pHrodo-labeled heat-inactivated *S. aureus*. The treatment chloroquine + heat-inactivated *S. aureus* +PG was included in the incubations. At 1 h after incubation, the cells were examined with confocal microscopy. The red spots showed the formation of phagolysosome. RAW264.7 cells and PG alone were used as controls. Scale bar, 10 μm (up) or 5 μm (down). (**D**) The detection of the total *S. aureus* using a pH-insensitive PCR. The RAW 264.7 cells were treated with PG/LPS and *S. aureus*. At 2 h after treatments, the cells were subjected to PCR with the *S. aureus nuc* gene-specific primers. M indicates the DNA marker. (**E**) The percentage of phagocytosed pHrodo-labeled inactivated *S. aureus* in RAW264.7 cells. The RAW264.7 cells were incubated with LPS or PG and pHrodo-labeled inactivated *S. aureus*. At 1 h after incubation, the cells were evaluated using flow cytometry. Non-treated cells and PG alone were used as controls. Statistically significant differences between treatments were indicated with asterisks (**P < 0.01).

To evaluate the phagocytic activity of RAW264.7 cells against *Staphylococcus aureus*, the bacteria were inactivated and then subjected to phagocytosis assay in RAW264.7 cells. The use of inactivated bacteria in the evaluations of phagocytic activity of macrophages could exclude the effects of bacterium infection on phagocytosis. The heat-inactivated *S. aureus* was engulfed, but not digested by RAW264.7 cells (Fig. 1A,B). We hypothesized that the receptors on the surface of macrophages were not activated to recognize and subsequently induce the digestion of *S. aureus*.

In order to activate phagocytosis of RAW264.7 cells against *S. aureus*, lipopolysaccharide (LPS) and peptidoglycan (PG) were added into cells respectively. The results showed that the heat-inactivated *S. aureus* was completely phagocytosed by RAW264.7 cells induced with PG (Fig. 1A,B). However, LPS did not induce the complete phagocytosis of inactivated *S. aureus* (Fig. 1A,B). After PG treatment, we determined that the RAW264.7 cells became acidic (Fig. 1C), which is an indicator of phagosome maturation. However, after LPS treatment, the pH of the RAW264.7 cells was similar to the un-treated cells (Fig. 1C). As a control, we treated RAW264.7 cells with PG alone (no *S. aureus*) and found no change in pH (Fig. 1A–C).

To explore the effects of specific inhibitors on the phagosomal acidification, chloroquine was used in the phagosomal acidification assays. It was found that phagosomal acidification was inhibited after treatment with chloroquine (Fig. 1C). We also wanted to detect the total *S. aureus* with a pH-independent method, and PCR using the *S. aureus nuc* gene was conducted. The results showed that *S. aureus* was detected in the LPS-treated RAW 264.7 cells, but not in the PG-treated cells (Fig. 1D), confirming our previous results (Fig. 1A–C). The percentage of phagocytosed *S. aureus* was significantly higher (*P* < 0.01) in PG-treated cells than in any of the other treatments (Fig. 1E). Taken together, these findings indicated that PG could efficiently activate the phagocytic activity of phagocytes against *S. aureus*.

### The JAK-STAT pathway is altered after PG and *S. aureus* treatment

**Figure 2:**
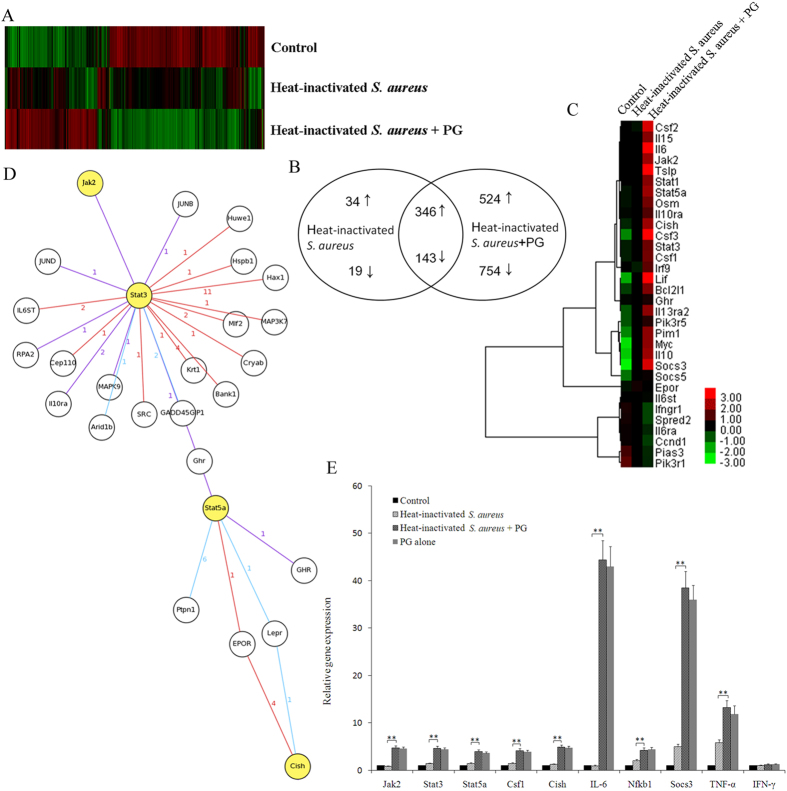
Analysis of signaling pathways required for the phagosome maturation. (**A**) The gene expression profiles were conducted with a DNA microarray using RAW264.7 cells treated with heat-inactivated *S. aureus* or heat-inactivated *S. aureus* and PG. Non-treated RAW264.7 cells were used as a control. Red indicated up-regulation of gene expression and green showed down-regulation of gene expression. (**B**) Venn diagram of differentially expressed genes. The differentially expressed genes were evaluated using DNA microarray data. The numbers represented the genes up-regulated or down-regulated (more than twofold) compared with the control. Arrows indicated up- or down-regulated genes. (**C**) Hierarchical cluster analysis of DNA microarray data. Clustering of 32 genes in JAK-STAT pathway in response to the challenge of heat-inactivated *S. aureus* or heat-inactivated *S. aureus* and PG. (**D**) The biological processes mediated by the differentially expressed genes. The 524 up-regulated genes in RAW264.7 cells treated with heat-inactivated *S. aureus* and PG were analyzed. The JAK-STAT pathway represented the key pathway. The red, blue and purple lines indicated the IntAct, MINT and DIP databases, respectively. The numbers indicated a protein’s interaction degree. (**E**) The expressions of genes from JAK-STAT pathway in RAW264.7 cells in response to PG challenge. At 1 h after challenge with heat-inactivated *S. aureus*, heat-inactivated *S. aureus* + PG and PG alone, the expression profiles of Jak2, Stat3, Stat5a, Cish, Csf1, IL-6, Socs3, Nfkb1, TNF-α and IFN-γ genes in RAW264.7 cells were characterized by quantitative real-time PCR. The GAPDH gene was used as a control. Statistically significant differences between treatments were indicated with asterisks (**P < 0.01). Control, RAW264.7 cells only.

To determine which genes and pathways were involved in phagosome maturation in RAW264.7 cells, a genome-wide analysis using oligonucleotide microarray was conducted after the cells were incubated with PG and inactivated *S. aureus*. The gene expression profiles of RAW264.7 cells treated with inactivated *S. aureus* and PG or inactivated *S. aureus* differed from untreated cells) (Fig. 2A). This finding suggested that PG could induce various receptors and pathways.

The microarray revealed that 380 and 162 genes were significantly (*P* < 0.01) up-regulated and down-regulated, respectively in the RAW264.7 cells treated with heat-inactivated *S. aureus*, compared with the untreated control (Fig. 2B). After *S. aureus* and PG treatment, 870 and 897 genes were significantly up-regulated and down-regulated (*P* < 0.01), respectively (Fig. 2B). The comparison analysis indicated that 1278 genes were differentially expressed in the RAW264.7 cells treated with inactivated *S. aureus* and PG (Fig. 2B). Among these genes, 524 genes were up-regulated in response to the PG treatment, suggesting that PG played an important role in the phagocytosis against *S. aureus*.

The gene network analysis of 524 up-regulated genes showed that the JAK-STAT pathway represented the key pathway in the RAW264.7 cells treated with inactivated *S. aureus* and PG (Fig. 2C,D). This indicated that PG might induce the JAK-STAT pathway, which may be necessary in phagosome maturation. Genes in the JAK-STAT pathway that were significantly (*P* < 0.01) up-regulated were *JAK2*, *Stat1*, *Stat3*, *Stat5a*, *IL-6*, *IL-10*, *IL10ra*, *IL13ra2*, *IL-15*, *IRF9*, *Csf1*, *Csf3*, *Cish*, *Lif*, *Socs3*, *Myc*, *Pim1* and *Tslp*, but *Ifngr1*, *Spred2*, *Il6ra*, *Ccnd1*, *Pias3* and *Pik3r1* were significantly (*P* < 0.01) down-regulated (Fig. 2C).

To confirm the gene expression data from the microarray, the quantitative real-time PCR was performed. The relative expressions of *JAK2*, *Stat3*, *Stat5a*, *Cish*, *Csf1*, *IL-6*, *Socs3* and *Nfkb1* genes was significantly (*P* < 0.01) up-regulated in the RAW264.7 cells after treatment with inactivated *S. aureus* and PG or PG alone compared to cells treated with inactivated *S. aureus* alone (Fig. 2E). In addition, the expression of *TNF* (tumor necrosis factor)-α was significantly (*P* < 0.01) up-regulated in cells treated with inactivated *S. aureus* and PG, whereas the expression of IFN (Inteferon)-γ was not affected (Fig. 2E). These findings demonstrated that PG could activate the JAK-STAT signaling pathway in the phagocytosis against inactivated *S. aureus*.

### The JAK-STAT pathway is necessary for phagosome maturation

**Figure 3:**
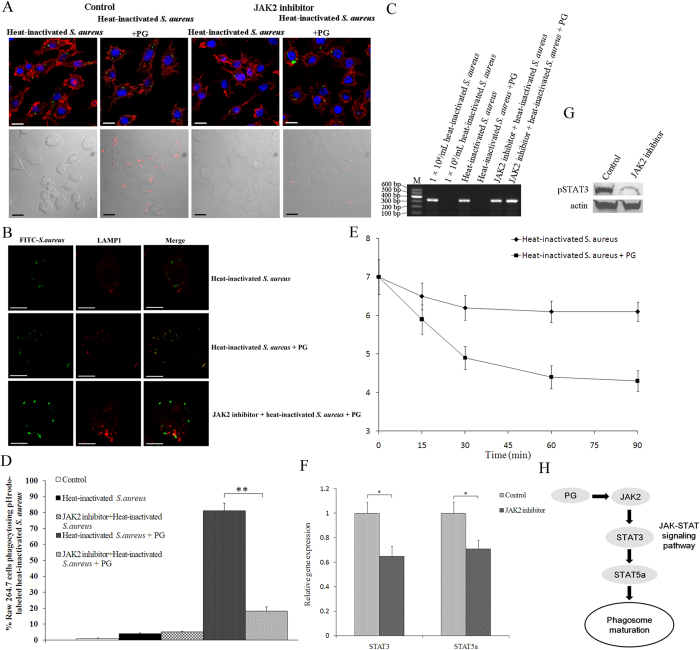
The role of JAK-STAT pathway in phagosome maturation. (**A**) Confocal microscopy of phagocytosis of inactivated *S. aureus* by RAW264.7 cells. RAW264.7 cells were inoculated with the JAK2 inhibitor, followed by the incubation with the FITC-labeled inactivated *S. aureus* (up) or the pHrodo-labeled *S. aureus* (down) in the presence or absence of PG. One hour later, the cells were examined with confocal microscopy. The cells without the JAK2 inhibitor were used as controls. Scale bar, 10 μm. (**B**) Localization of LAMP1 protein expressed in RAW 264.7 cells. The green points indicated the FITC-labeled *S. aureus*. Scale bar, 10 μm. (**C**) The evaluation of the total *S. aureus* with a pH-independent strategy. The RAW 264.7 cells were treated with PG, the JAK2 inhibitor and *S. aureus*. At 2 h after treatments, the total *S. aureus* in cells were detected with PCR using the *S. aureus nuc* gene-specific primers. M indicates the DNA marker. (**D**) The percentage of phagocytosed pHrodo-labeled inactivated *S. aureus* in RAW264.7 cells. The number of cells phagocytosing the pHrodo-labeled inactivated *S. aureus* was quantified using flow cytometry at 1 h after inoculation of *S. aureus*. The cells without the JAK2 inhibitor were used as controls. Statistically significant differences between treatments were indicated with asterisks (**P < 0.01). (**E**) The measurement of pH values. The phagosome pH was measured in RAW 264.7 cells using the dual-labeled *S. aureus* ratiometric assay. (**F**) Effects of the inhibition of JAK2 activity on expressions of genes of JAK-STAT pathway. RAW264.7 cells were treated with the JAK2 inhibitor. Six hours later, the expressions of STAT3 and STAT5a genes were evaluated using real-time PCR. Statistically significant differences between treatments were indicated with asterisks (*P < 0.05). (**G**) The influence of the inhibition of JAK activity on the STAT3 phosphorylation. The JAK2-inhibitor-treated RAW264.7 cells were analyzed with Western blot using phosphorylated STAT3-specific antibody. Actin was used as a control. (**H**) The model of PG-triggered JAK-STAT signaling pathway in the phagosome maturation.

To determine the role of JAK-STAT pathway in the maturation of phagosomes, RAW264.7 cells were treated with the JAK2 inhibitor, followed by the phagocytosis assays using pHrodo-labeled inactivated *S. aureus*. Phagosome maturation was evident after the inoculation of PG and inactivated *S. aureus* but not in the control (inactivated *S. aureus* alone) (Fig. 3A). After the addition of the JAK2 inhibitor to cells inoculated with inactivated *S. aureus* and PG, phagosome maturation was abolished (Fig. 3A), indicating that the PG-triggered JAK-STAT signaling pathway was essential for the phagosome maturation during phagocytosis. For colocalization of phagosome and *S. aureus*, we labeled LAMP1 (Lysosomal-associated membrane protein 1) with PE-modified rat antibody which shows the red fluorescence. As expected, the LAMP1 proteins localized to most lysosomes in cells treated with PG and inactivated *S. aureus* but not in cells treated with inactivated *S. aureus* alone (Fig. 3B). After the addition of the JAK2 inhibitor, the LAMP1 did not significantly localize with FITC-labeled inactivated *S. aureus* (Fig. 3B). We also determined the presence of *S. aureus* by PCR. *S. aureus* was detected in the RAW264.7 cells treated with the JAK2 inhibitor (Fig. 3C), which further confirmed our previous findings. The percentage of phagocytosed pHrodo-labeled inactivated *S. aureus* in PG-induced RAW264.7 cells was significantly higher (*P* < 0.01) than in PG-induced cells treated with the JAK2 inhibitor (Fig. 3D). The pH detection of phagosomes also showed that phagosomes acidified to pH < 5.0 by 30min in PG-treated cells (Fig. 3E). These findings demonstrate that the JAK-STAT pathway was required for phagosome maturation.

Inhibition of JAK2 activity led to a significant decrease in *Stat3* and *Stat5a* gene expression in RAW264.7 cells (Fig. 3F). The protein expression of phosphorylated STAT3, the active form of STAT3, was significantly (*P* < 0.01) decreased after JAK2 inhibition (Fig. 3G), indicating that JAK2 was required for STAT3 phosphorylation. Considering all the above data, the PG induced JAK-STAT signalling pathway played an essential role in phagosome maturation (Fig. 3H).

### Phagosome maturation requires the JAK-STAT pathway after inoculation with live *S. aureus*

**Figure 4:**
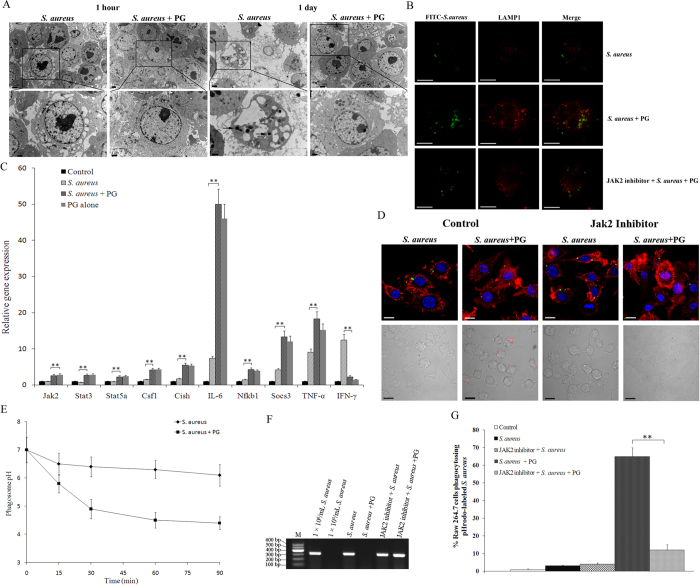
The effect of PG-induced JAK-STAT pathway on phagocytosis against live *S. aureus*. (**A**) RAW264.7 cells were activated by PG, followed by incubation with live *S. aureus*. One hour or one day later, the cells were examined with transmission electron microscope. The black arrows indicate the intracellular *S. aureus.* Lane headings indicated the treatments. The box indicated the enlarged image. Scale bar, 2 μm (up) or 1 μm (down). (**B**) Localization of LAMP1 expressed in RAW 264.7 cells. The green points showed the FITC-labeled *S. aureus*. Scale bar, 10 μm. (**C**) The expressions of genes from the JAK-STAT pathway in RAW264.7 cells in response to PG challenge. At 1 h after challenge with live *S. aureus* or live *S. aureus* + PG or PG alone, the expression profiles of Jak2, Stat3, Stat5a, Cish, Csf1, IL-6, Socs3 and Nfkb1, TNF-α and IFN-γ genes in RAW264.7 cells were examined with real-time PCR. The GAPDH gene was used as a control. Statistically significant differences between treatments were indicated with asterisks (**P < 0.01). Control, RAW264.7 cells only. (**D**) RAW264.7 cells were inoculated with the JAK2 inhibitor, followed by incubation with the FITC-labeled *S. aureus* (up) or the pHrodo-labeled *S. aureus* (down) in the presence or absence of PG. One hour later, the cells were examined with confocal micrscopy. The cells without the JAK2 inhibitor were used as controls. Scale bar, 10 μm. (**E**) The pH values in macrophages. The phagosome pH was measured in RAW 264.7 cells using the dual-labeled *S. aureus* ratiometric assay. (**F**) The examination of the total *S. aureus* in RAW 264.7 cells. The RAW 264.7 cells were treated with PG, the JAK2 inhibitor and *S. aureus*. At 2 h after treatments, the total *S. aureus* in cells were detected with PCR using the *S. aureus nuc* gene-specific primers. M indicates the DNA marker. (**G**) The percentage of phagocytosed pHrodo-labeled *S. aureus* in RAW264.7 cells. The number of cells phagocytosed the pHrodo-labeled *S. aureus* was quantified using flow cytometry at 1 h after inoculation of *S. aureus*. The cells without the JAK2 inhibitor were used as controls. Statistically significant differences between treatments were indicated with asterisks (**P < 0.01).

We next determined the effects of PG on phagosome maturation after RAW264.7 cells were inoculated with live *S. aureus*. Using transmission electron microscopy, we observed no bacteria in the cells at 1 hour after inoculation (Fig. 4A). At one day after inoculation, the cells treated with live *S. aureus* alone were dead and many bacteria were present (Fig. 4A). However, the cells treated with live *S. aureus* and PG grew normally and no bacteria were present (Fig. 4A). The LAMP1 proteins were colocalized with many lysosomes in cells treated with PG and *S.aureus* but not in cells treated with *S. aureus* alone (Fig. 4B). These data suggested that PG could activate phagosome maturation in order to phagocytose bacteria.

The quantitative real-time PCR was conducted to characterize the expression of key genes of JAK-STAT pathway. The results showed that the relative expressions of *JAK2*, *Stat3*, *Stat5a*, *Cish*, *Csf1*, *IL-6*, *Nfkb1*, *Socs3* and *TNF-α* were significantly (*P* < 0.01) up-regulated and IFN-γ expression was significantly (*P* < 0.01) down-regulated in the RAW264.7 cells treated with live *S. aureus* and PG or PG alone compared to cells treated with live *S. aureus* alone (Fig. 4C). These findings were similar to those observed when cells were inoculated with inactivated *S. aureus*.

The role of JAK-STAT pathway was characterized by inhibiting JAK2 in RAW264.7 cells incubated with the pHrodo-labeled live *S. aureus*. Using confocal microscopy, we observed phagosome maturation at one hour after inoculation with PG and live *S. aureus* (Fig. 4D). The pH detection of phagosomes also showed that phagosomes acidified to pH < 5.0 by 30min in PG-treated cells (Fig. 4E). Western blot showed the significant knockdown of STAT3 in JAK inhibitor treated RAW cells (Fig. 3G). As the control, the expression of β-actin was detected by anti-actin antibodies and showed no difference among all treatments (Fig. 3G). However, the JAK2 inhibitor inhibited the phagosome maturation even in the presence of PG (Fig. 4B,D), indicating that the JAK-STAT pathway plays an important role in phagosome maturation. The PCR result showed that *S. aureus* was detected in the JAK2 inhibitor-treated RAW264.7 cells (Fig. 4F), suggesting that the PG-induced JAK-STAT pathway may be required to phagocytose live *S. aureus*. The percentage of phagocytosed pHrodo-labeled live *S. aureus* in PG-activated RAW264.7 cells was significantly higher (*P* < 0.01) than that in PG-treated cells treated with the JAK2 inhibitor (Fig. 4G). Taken together, PG can induce the JAK-STAT pathway, which plays an important role in phagosome maturation.

## Discussion

As reported, the pathogenic microbes are internalized and rapidly delivered into a mature phagolysosome where they are killed and degraded^6^,^7^. A study shows that the bacterial killing occurs in *S. aureus*-infected neutrophils without apparent degradation of bacteria^20^, suggesting that lack of degradation of bacteria may not indicate defects in phagosome maturation or bacterial killing. However, the phagosome maturation is one of the key steps of phagocytosis. It is found that numerous pathogens have evolved complex mechanisms to manipulate host intracellular organelles in order to survive^21^. *S. aureus*, an important pathogen in humans, can invade host cells and persist intracellularly in cell culture models^22^. This strategy allows *S. aureus* to evade the host’s professional phagocytes and extracellular antibiotics, which promotes ongoing infection^23^. Likewise, *S. aureus* can survive in human epithelial cells for prolonged periods of time^24^. It is believed that phagocytosis is required for the host defense against Gram-negative microbes as well as Gram-positive pathogens^6^,^7^. However, the phagocytosis of *S. aureus* has not been intensively investigated.

In this study, PG induced the JAK-STAT signaling pathway to activate phagosome maturation in order to eradicate both inactivated and live *S. aureus*. Our data indicated that the expressions of cytokines in RAW264.7 cells were significantly up-regulated in response to exogenous PG in the presence or absence of inactivated *S. aureus*. This suggests that PG, not heat-inactivated *S. aureus*, was responsible for the secretion of cytokines from the activated phagosomes. This finding was consistent with other data^17^,^18^. RAW264.7 cells do not express the apoptotic speck protein, which is one of the key molecules in the inflammasome complex. Thus *S. aureus* peptidoglycan must be particulate and internalized via phagocytosis to activate the NLRP3 inflammasomes and IL-1β secretion^17^. Although RAW264.7 cells will not undergo NLRP3-dependent pyroptosis in response to *S. aureus*^25^, PG stimulation could induce effective phagocytosis to clear *S. aureus*. In this context, our study presented novel aspects of PG and the JAK-STAT signaling pathway in phagocytosis of Gram-positive bacteria.

The Janus kinases (JAKs) play critical roles in several important intracellular signaling pathways, including the JAK-STAT pathway and the mediation of cytokine signaling^26^,^27^. And 40 cytokine receptors may perform signal transductions through the combinations of four JAK and seven STAT family members^28^. JAK1, JAK2, JAK3 and Tyk2 are involved in cell growth, survival, development, and differentiation of cells^28^. JAK2, a member of the JAKs family of protein tyrosine kinases, is an important intracellular mediator of cytokine signaling^29^. JAK2 can induce the activation of macrophages, the inflammatory response and the inhibition of apoptosis^30^,^31^. It has been reported that protein A from *S. aureus* is endocytosed by airway epithelial cells and subsequently activates the JAK-STAT signaling pathway^32^. Our data showed that PG from Gram-positive bacteria could activate the JAK-STAT pathway, leading to phagosome maturation. It is evidenced that *S. aureus* can promptly escape from the endosomes/phagosomes and proliferate within the host cytoplasm and quickly leads to host cell death^33^,^34^. These data have provided evidence to test potential therapeutic strategies to reduce pathogenicity of Gram-positive bacteria such as *S*. *aureus*.

## Materials and Methods

### Strain and cell culture

The *Staphylococcus aureus* strain ATCC25923, widely used for phagpocytosis assays, was obtained from Honghui hospital (Hangzhou, China). *S. aureus* was grown overnight in Columbia medium (Oxoid, UK) with 2% NaCl at 37 °C. *Mus musculus* RAW264.7 cells (Cell Bank of Chinese Academy of Science, China) were cultured in RPMI 1640 media (Hyclone, USA) supplemented with 10% heat-inactivated/refiltered fetal bovine serum (FBS) (Gibco, USA), 10 mM HEPES (4-(2-hydroxyethyl)-1-piperazineethanesulfonic acid) (Invitrogen, USA), 0.11 mg/mL sodium pyruvate (Sigma, USA), 0.002 M L-glutamine (Sigma, USA) and pen/strep (1 mg/mL, 100 U/mL) (Invitrogen, USA).

### Phagocytosis assay

The cultures of *S. aureus* were suspended in phosphate-buffered saline (PBS) and inactivated by heat at 85 °C for 60 min. Then the inactivated or live *S. aureus* were added into RAW264.7 cells at a density of 5 × 10^6^/mL, followed by incubation for 1 h. To determine whether lipopolysaccharide (LPS) or peptidoglycan (PG) enhanced the efficient phagocytosis, RAW264.7 cells were plated at a concentration of 1 × 10^6^ cells per plate in six-well plates and were allowed to adhere for at least 30 min. LPS or PG (Sigma, USA) was sonicated for 1 h and then added to each well at a concentration of 1 ng/ml for LPS or 10 μg/ml for PG. The cells containing LPS or PG were incubated for 5 h. Subsequently the cells were treated with the heat-inactivated or live *S. aureus*. At different time after inoculation of bacteria, RAW264.7 cells were collected for later use.

### The detection of the total *S. aureus* in Raw264.7 cells

Raw264.7 cells were treated with *S. aureus* and/or PG, LPS and the JAK2 inhibitor. At 2 h after treatments, the total DNA in Raw264.7 cells was extracted using QlAamp DNA Mini Kit (Qiagen, Germany). Then PCR was conducted using *S. aureus nuc* gene-specific primers (5′-GCGATTGATGGTGATACGGTT-3′ and 5′-G CGTTGTCTTCGCTCCAAAT-3′) according to the previous study^35^.

### Transmission electron microscopy

RAW264.7 cells were treated as previously described^36^. Sections were cut in a Reichert Ultracut OMU3 microtome (Leica, Germany) at 100 nm thickness, followed by staining with uranyl acetate/70% methanol. The images were obtained on a Hitachi 7650 transmission electron microscope (Hitachi, Japan) operating at 70 kV.

### Confocal microscopy

RAW264.7 cells were plated in 6-well plastic tissue culture plates in 1 ml of Schneider’s medium with 10% FBS (Gibco, USA). The inactivated *S. aureus* was labeled with FITC (Invitrogen, USA) and then added into RAW264.7 cells at a density of 5 × 10^6^/mL. The cells were incubated for different time at 37 °C. After washes with PBS, the cells were transferred to poly-prep glass slides (Sigma, USA). The filamentous actin and DNA of RAW264.7 cells were separately stained with rhodamine phalloidin (Invitrogen, USA) and DAPI (Sigma, USA). The LAMP1 (Lysosomal-associated membrane protein 1) proteins were labeled with PE Rat anti-Mouse CD107a (BD Pharmingen, USA). Fluorescent images were taken with a Zeiss Laser scanning systems LSM 510 Meta (Carl Zeiss, Germany). Images were processed using Zeiss LSM Image Examiner Version software.

### The measurement of phagosomal acidification

To evaluate the phagosomal acidification, the *S. aureus* was labeled with pHrodo dye (Invitrogen, USA), which could dramatically increases the fluorescence as the microenvironment of its surroundings becomes more acidic^37^. RAW264.7 cells (1 × 10^6^/mL) were incubated with pHrodo-labeled bacteria for 1 h at 37 °C, followed examination by confocal microscopy. To characterize the effects of specific inhibitors on the phagosomal acidification of macrophages, chloroquine was used (Shimada *et al.*, 2010). RAW264.7 cells were added with 200 μM of chloroquine (Sigma-Aldrich, USA). Thirty min later, the cells were treated with pHrodo-labeled *S. aureus* for 1 h at 37 °C. Subsequently the cells were examined with confocal microscopy.

### The activation of RAW264.7 cell phagocytosis with LPS or PG

To activate the phagocytic activity of RAW264.7 cells, lipopolysaccharide (LPS) or peptidoglycan (PG) was added into RAW264.7 cells, followed the detection of phagocytosis against *S. aureus*. LPS and PG were purchased from Sigma (USA). RAW264.7 cells were treated as previously described^38^. Subsequently the heat-inactivated *S. aureus* was added into RAW264.7 cells. As controls, PG only and LPS only were included in the assays. At different time, the cells were collected and examined with transmission electron microscope and confocal microscopy.

### The analyses of mRNA expression using oligonucleotide arrays and biological pathways

Total RNAs were extracted from RAW264.7 cells (1×10^6^) using Trizol reagent (Invitrogen, USA) according to the manufacturer’s instructions. The total RNAs were then subjected to the Affymetrix Mouse Genome 430 2.0 array GeneChip (CapitalBio Corp., CA, USA) for gene expression analysis. Microarray data were analyzed using Bio MAS (molecule annotation system) 3.0 software (CapitalBio Corp.). Using the criterion of cutoff limitation as a fold change ≥2 or ≤0.5 and q-value ≤5%, differentially expressed genes were screened and clustered. Based on DNA microarray, the differently altered genes were subjected to biological pathway analysis. The selected genes were analyzed in the context of Kyoto Encyclopaedia of Genes and Genomes (KEGG) using MAS 3.0 software.

### Quantitative real-time RT–PCR

Total RNAs were extracted from RAW264.7 cells (1 × 10^6^) with Trizol reagent (Invitrogen, USA) according to the manufacturer’s manual. Then the total RNAs were reversely transcribed to synthesize cDNAs using reverse transcriptase M-MLV (Takara, Japan). Quantitative real-time PCR was performed using gene-specific primers and TaqMan probes (Table 1). Relative gene expression levels were calculated according to the previous method^39^, using GAPDH as an internal control. Real-time PCR amplification reactions were carried out in a final volume of 20 μl, which contained 10 μl Premix Ex Taq (TaKaRa, Japan), 1 μl diluted cDNA template, 7.2 μl dH_2_O, 0.3 μl of each TaqMan probe and 0.4 μl of each primer. PCR conditions were as follows, 95 °C for 30 seconds, followed by 50 cycles of 95 °C for 5 seconds and 60 °C for 30 seconds.

### The Inhibition assay of JAK2 activity

To reveal the role of JAK-STAT signaling pathway in phagocytosis, the activity of the key enzyme JAK2 in this pathway was inhibited with the JAK2 inhibitor 2-(1, 1-Dimethylethyl)-9-fluoro-3, 6-dihydro-7H-benz[h]-imidaz[4, 5-f]isoquinolin-7-one (Millipore, USA) at 2 nM. RAW264.7 cells (1 × 10^6^/mL) were incubated with the JAK2 inhibitor for 6 h. Subsequently the sonicated PG (Sigma, USA) was added into the cells at a concentration of 1 μg /mL. One hour later, the cells were inoculated with heat-inactivated *S. aureus* at a density of 5 × 10^6^/mL. At 1 h after incubation, the cells were examined using confocal microscopy and flow cytometry. The RAW264.7 cells without the JAK2 inhibitor were used as controls.

### Western blotting

Proteins were separated by SDS-PAGE and then transferred to a polyvinylidene fluoride (PVDF) membrane (Millipore, USA). The membrane was blocked with blocking buffer (5% milk in Tris-buffered saline and Tween-20) for 1 h at room temperature. Subsequently the membrane was incubated with a primary antibody at 4 °C overnight. After three washes with Tris-buffered saline, the membrane was incubated with alkaline phosphatase-conjugated secondary antibody (Roche, Switzerland) for 2 h at room temperature. The membrane was detected with BCIP/NBT substrate (Sangon Biotech, Shanghai, China). The primary antibodies were purchased from Cell Signal Technology (USA).

### pH detection of phagosomes

The phagosomal pH was assayed according to the previous described method^19^. Briefly RAW264.7 cells were incubated with FITC (pH-sensitive)-labeled or Alexa Fluor 647 (pH-insensitive)-labeled *S. aureus* at low MOI (multiplicity of infection) (≤10) for 30 min on ice, allowing the synchronization of bacteria binding onto cells. Subsequently the cells were incubated for the indicated times at 37 °C. After washes twice with ice-cold PBS/EDTA (eathylene diamine tetraacetic acid), the cells were detached and immediately analyzed by flow cytometry to determine the mean fluorescent index emission between FITC and Alexa Fluor 647. To calculate pH using the ratiometric assay, values were compared with a standard curve obtained by resuspending and permeabilizing the cells that had phagocytosed bacteria for 2 h in buffers at a fixed pH (ranging from pH 3.5 to 8). The cells were immediately analyzed by flow cytometry to determine the emission ratio of the two fluorescent dyes at each pH.

### Statistical analysis

The data from three independent experiments were analyzed by one-way analysis of variance to calculate the means and standard deviations of the triplicate assays. The significant difference between treatments was determined by Student’s t-test.

## Additional Information

**How to cite this article**: Zhu, F. *et al.* Role of JAK-STAT signaling in maturation of phagosomes containing *Staphylococcus aureus*. *Sci. Rep.* **5**, 14854; doi: 10.1038/srep14854 (2015).

## Figures and Tables

**Table 1:** Sequences of primers and TaqMan probes used for quantitative real-time PCR.

Gene	Forward primer (5′-3′)	Reverse primer (5′-3′)	TaqMan probe (5′-3′)
GAPDH	caatgtgtccgtcgtggatct	gtcctcagtgtagcccaagatg	cgtgccgcctggagaaacctgcc
Jak2	cttccacatagacgagtcaacca	catcaagcagaggagcttcagc	atctgtaggttctgctgctgccact
Stat3	ccgatgcctgtgggaagagtc	tgtcactacggcggctgttg	cctccagacggcagccacggca
Stat5a	gcagtcctggtgtgagaagc	tgagatgatgtccgtgatggtg	acctcagccagcatctcctccacg
Cish	ccctgcctatgtctaagcaagat	gccaccagacggttgatgac	cctagtgactcggtgctgcctatcc
Socs3	ccactacatgccgcctccag	cggctcagtaccagcggaatc	tctttgccacccacggaaccctcg
Csf1	cgctgcccttcttcgacatg	ccttcaggtgtccattcccaatc	cggctgctgctggtctgtctcctc
IL-6	ctctgcaagagacttccatccag	tctcctctccggacttgtgaag	caccagcatcagtcccaagaag
Nfkb1	agtgcaaaggaaacgccagaag	gccagggcttccggtactc	tccgccaccgccactaccga
TNF-α	atgttgtagcaaaccctgaagct	attggccaggagggcatt	Actccaatggctgagccgacgtg^43^
IFN-γ	tggtgggcctcttttcttagatat	agaaggagacaatttggctttga	Ttgaagaactggagagaggagagtgacaaaaaaa^43^
